# Comparison of rural practice in relation to upper gastrointestinal endoscopy

**DOI:** 10.12669/pjms.37.4.4397

**Published:** 2021

**Authors:** Kazuhiko Kotani

**Keywords:** Gastrointestinal endoscopy, Gastric cancer, Gastroenterologist, Rural health, Rural medicine

*Dear Editor*,

We read with great interest the Kamran study on the findings of clinical practice with upper gastrointestinal endoscopy (GIE) in a rural hospital in Pakistan.^1^ Worldwide, there have been few data specific to rural practice in relation to GIE; thus, the findings from such studies provide valuable information to consider in the clinical picture of gastrointestinal pathologies among rural populations and the provision of rural healthcare systems. To consider this, an understanding of comparative situations across many countries is useful. We would like to describe additional comments on the Kamran study in comparison to the Japanese situation, as we recognized a clear difference in rural practice in relation to GIE between the respective countries.

The first comment is from a pathological viewpoint. The difference in gastrointestinal pathologies between countries is well-known; namely, the highest incidence of gastric cancer is shown in the general Japanese population. In contrast, the incidence in the general Pakistani population is low.^2^ While gastric cancer is commonly seen in the rural hospitals of Japan, the Kamran study demonstrates no cases in which gastric cancer was detected by GIE.^1^ Thus, the difference in the clinical picture of gastrointestinal pathologies between Pakistan and Japan even appears in rural populations. The reasons for the between-country difference in the incidence of gastric cancer remain unknown.^2^ As rural populations often share particular cultural, lifestyle, and environmental factors, larger and more detailed comparative studies (between rural and urban areas as well as between countries) that investigate etiologic factors, with the inclusion of gastric findings of more rural populations based on the full use of GIE (as a direct diagnostic tool of diseases), may provide some hints as to the reasons.

The second comment is from a socio-medical viewpoint. There are differences in the development of rural healthcare systems with GIE, followed by the incidence of gastric cancer; for instance, under the government-led policies, in Japan, screening for gastric cancer using GIE has been conducted nationwide. Almost all public hospitals built in rural municipalities have GIE equipment.^3^ The rural healthcare system is associated with the clinical practice of doctors working in rural hospitals. Among doctors graduating from Jichi Medical University, which was launched to produce rural doctors in Japan, gastroenterology is the most common board-certified subspecialty field. In recent days, not only screening but also treatments that require a high degree of skill, such as gastric submucosal dissection, using GIE, are performed in rural hospitals. This Japanese experience implies that the epidemiology of pathologies and the government strategy are involved in the arrangement of the rural healthcare system, including the manner of practice by doctors.

The accumulation of more knowledge on rural practice in relation to GIE, as is provided by the Kamran study,^1^ is necessary for comparing the situations of many countries. Ultimately, this would be an activity to resolve the issues of health inequality in rural areas.

***Response from the authors:*** We appreciate the valuable comments of Prof. Kazuhiko Kotani on this original article. This was actuality a retrospective study which primarily aimed to look at the gross pathological findings in patients undergoing upper gastro-intestinal endoscopy in a rural population cohort, however histopathological findings were not assessed. In our study, we found that around 0.8% of patients had gastric growth on endoscopic examination (either benign or malignant). Having said that, this prevalence is much less than what has been reported from Japan^1^.

The World Bank ranks Pakistan among the lower-middle-income countries, based on the gross domestic product (GDP) per capita. Unlike Japan, the currently existing healthcare system in Pakistan is inadequate with regards to performing regular cancer screening in rural areas. In our opinion, this uphill task can be achieved by effective public and private sector partnership so that people in these underserved areas may have access to better healthcare.

**REFERENCES**

1. Hamashima C. Current issues and future perspectives of gastric cancer screening. World J Gastroenterol. 2014;20(38):13767-13774. doi:10.3748/wjg.v20.i38.13767
